# Identification of a β-Carboline Alkaloid from Chemoselectively Derived Vanilla Bean Extract and Its Prevention of Lipid Droplet Accumulation in Human Hepatocytes (HepG2)

**DOI:** 10.3390/molecules28248024

**Published:** 2023-12-09

**Authors:** Dya Fita Dibwe, Nire Takeishi, Saki Oba, Akiko Sakurai, Toshihiro Sakurai, Takayuki Tsukui, Hitoshi Chiba, Shu-Ping Hui

**Affiliations:** 1 Faculty of Health Sciences, Hokkaido University, Kita-12, Nishi-5, Kita-Ku, Sapporo 060-0812, Japan; dibwedf@hs.hokudai.ac.jp (D.F.D.); sakura@hs.hokudai.ac.jp (T.S.); 2 Graduate School of Health Sciences, Hokkaido University, Kita-12, Nishi-5, Kita-Ku, Sapporo 060-0812, Japan; n10461991724nasukakstg@docomo.ne.jp (N.T.); saki.oba.h@gmail.com (S.O.); sakurai.akiko.p6@elms.hokudai.ac.jp (A.S.); 3 Department of Nutrition, Sapporo University of Health Sciences, Nakanuma Nishi-4-3-1-15, Higashi-Ku, Sapporo 007-0894, Japan; tsukui@sapporo-hokeniryou-u.ac.jp (T.T.); chiba-h@sapporo-hokeniryou-u.ac.jp (H.C.)

**Keywords:** functional foods, bean extracts, bioactive alkaloids, lipid droplet accumulation inhibition, lipidomics, neutral lipids, triacylglycerols, Orbitrap LC/MS

## Abstract

Targeting bioactive compounds to prevent lipid droplet accumulation in the liver, we explored an antioxidative extract from vanilla bean (*Vainilla planifolia*) after chemo-selective derivatization through heating and acid modification. The chemical analysis of vanilla bean extract through chemoselective derivatization resulted in the identification of sixteen compounds (**34**–**50**) using LC-MS/MS analysis. A β-carboline alkaloid with a piperidine C-ring and a vanillin moiety at C-1 (**34**) was identified by molecular networking and diagnostic fragmentation filtering approaches. β-carboline alkaloid **34** exhibited significant inhibitory activity of lipid droplet accumulation (LDAI) in oleic acid-loaded hepatocellular carcinoma HepG2 cells. The LDAI activity was associated with both activation of lipolysis and suppression of lipogenesis in the cells. The study indicates that crude plant extracts, following chemoselective derivatization, may contain bioactive compounds that could be beneficial in preventing hepatosteatosis and could serve as a source of lead compounds for drug development. This approach may be useful to investigate other mixtures of natural products and food resources.

## 1. Introduction

Natural products, such as alkaloids, continue to play a significant role in the development of novel therapeutic agents in current century. Consequently, the diverse structures and complex carbon skeletons of natural products have led to their extensive use in drug discovery [1,2,3]. However, the isolation process is a limiting factor in the discovery of new compounds because it relies on extraction and chromatographic separation, which have a limited ability to purify and concentrate the minor components of a biological extract [4]. The conversion of functional groups in bioactive molecules is a valuable method for enhancing the availability of secondary metabolites in natural extracts [5,6,7,8]. Recently, several chemical procedures, including ammonolysis, sulfonylation, bromination, ethanolysis, and epoxidation have been performed on plant extracts to obtain new and potentially bioactive compounds [8,9]. Chemoselective derivatization under heat and acidic conditions may induce the cleavage of glycoside phenolic compounds and increase the aglycone phenolic derivatives in the mixture, with the amelioration of the antioxidant activity of the modified crude extract, which may be useful as a food supplement. As an example, the process of chemical derivatization of natural extracts obtained from *Larrea divaricata* Cav. led to an enhanced antioxidant activity and protein-precipitating capacity [10]. Another possible parallel reaction that may occur is the conversion of bioactive phenolic derivatives, such as vanillin, to minor alkaloids in the modified extract, which are precursors for the production of alkaloid metabolites in crude extracts. Therefore, chemoselective derivatization of crude extracts offers opportunities for the production of new alkaloid scaffold molecules and lead compounds for further drug discovery and development. 

Alkaloids are the predominant biologically active molecules found in natural herbs and are the source of some of the most important drugs currently in the market. Previous studies on biosynthesis and the Maillard reaction suggest that tryptophan and an aldehyde metabolite are the precursor molecules of the β-carboline alkaloids such as flazin and their derivatives in some medicinal plants and plant food [11,12,13]. β-Carbolines (βCs) are naturally occurring bioactive alkaloids, whereas α-dicarbonyl compounds are reactive substances generated in foods and in vivo. They were also present in foods and formed during the heating processes. HET-βC appeared in foods as a racemic mixture of enantiomers, suggesting the same mechanism of formation as the synthetized product [14]. Moreover, vanillin and aromatic amino acids present in plant food under similar acidic conditions at high temperatures could form a β-carboline alkaloids with a C-1 vanillin moiety in the heated vanilla mixture in the presence of L-tryptophan [11]. A previous study identified flazin, a β-carboline alkaloid with a carboxy group at C-3 and furfuryl alcohol moiety at C-1, and its derivative with a piperidine C-ring, in oyster, a functional food extract [15]. Subsequently, flazin and its derivative showed low cytotoxicity and significant lipid droplet accumulation inhibition (LDAI) activity. In addition, they significantly ameliorated lipid droplet accumulation (LDA) by inhibiting the accumulation of triacylglycerol species in the cells, activating lipolysis, and suppressing lipogenesis. Flazin is an antioxidant activator that upregulates nuclear factor erythroid 2–related factor 2 (Nrf2) [15,16]. These findings suggest that β-carboline alkaloid derivatives may help prevent non-alcoholic fatty liver disease (NAFLD). 

Targeting specific classes of compounds in crude extracts is a promising method for drug discovery and development because it enables the direct production and identification of natural products including alkaloids from complex and modified mixtures using recent metabolomic MS/MS techniques [17]. Additionally, the direct analysis of metabolites in extracts of complex bioactive natural mixtures has received increased attention owing to advances in liquid chromatography/mass spectrometry techniques [17,18,19,20,21,22], helping the scientific community identify bioactive metabolites directly from extracts with the aim of discovering and developing natural drugs and food supplements. A small number of reports have been published on the chemoselective derivatization of natural crude extracts, including alkaloids, and NMR and LC-MS metabolomics of extracts is gaining more attention [14,23,24,25]. However, there is a lack of information on bioactive β-carboline containing a C-1 substituted vanillin moiety and its LDAI capacity. Therefore, this study aimed to perform chemical profiling of the chemoselective derivatization of vanilla bean extracts, investigate and characterize potential β-carboline alkaloids in modified crude extracts, and evaluate their capacity to prevent lipid droplet accumulation in human hepatocytes (HepG2). In this study, we modified the antioxidant extract of *Vinilla planifolia* by mimicking the conditions of thermal derivatization to diversify its secondary metabolites and to produce alkaloid derivatives in the modified crude extract. We analyzed the chemical profile to identify and characterize potential alkaloids using the GNPS molecular network, and evaluated the LDAI activity of the identified alkaloids. 

## 2. Results and Discussion

### 2.1. Antioxidant Activity, Total Polyphenol Content (TPC), and Metabolite Profiling and Fingerprinting of Selected Bean Extracts

#### 2.1.1. Antioxidant Activity

A significant proportion of nutraceuticals and functional foods are herb-based medicinal products. These products have been found to possess physiological benefits, which can be attributed to their antioxidant properties. It is widely recognized that antioxidants are effective in mitigating the action of free radicals, which are involved in the onset of many chronic diseases [26]. Beans are nutrient-rich foods containing various proteins and secondary metabolites that are beneficial for health. The present study determined the antioxidant capacity of selected bean extracts (BEs: BE1–BE8) using a DPPH radical scavenging assay. BE3, BE7, and BE8 were the most active, with values of 5.96 nmolTE/mg, 7.38 nmolTE/mg, and 7.60 nmolTE/mg, respectively (Table 1). These results suggest that these active bean extracts can be used as functional foods for the treatment of oxidative diseases and represent a potential source of primary and secondary bioactive metabolites [26]. In addition, the TPC of eight selected BEs samples (BE1–BE8) was examined. Among all the BEs tested, BE5 (59.7 μg/mg) and BE8 (60.8 μg/mg) have the highest phenolic content (Table 1), suggesting that these extracts may be a potential source of polyphenolic compounds [26]. This result indicates that the content of phenolic compound derivatives in BE8 may be associated with the observed DPPH activity. In addition, previous reports indicated that phenolic metabolites are involved in preventing metabolic diseases [27,28,29]. Thus, the increased phenolic content in BE8 might be associated with its antioxidant activity. Therefore, in this study, comprehensive metabolite profiling of all selected BEs was performed by ^1^H-NMR and LC-MS analyses using the GNPS-MN and DFF approaches (Supporting Information: Materials and Methods).

#### 2.1.2. Proton Nuclear Magnetic Resonance Profiling and Fingerprinting of Selected Bean Extracts

**Figure 1:**
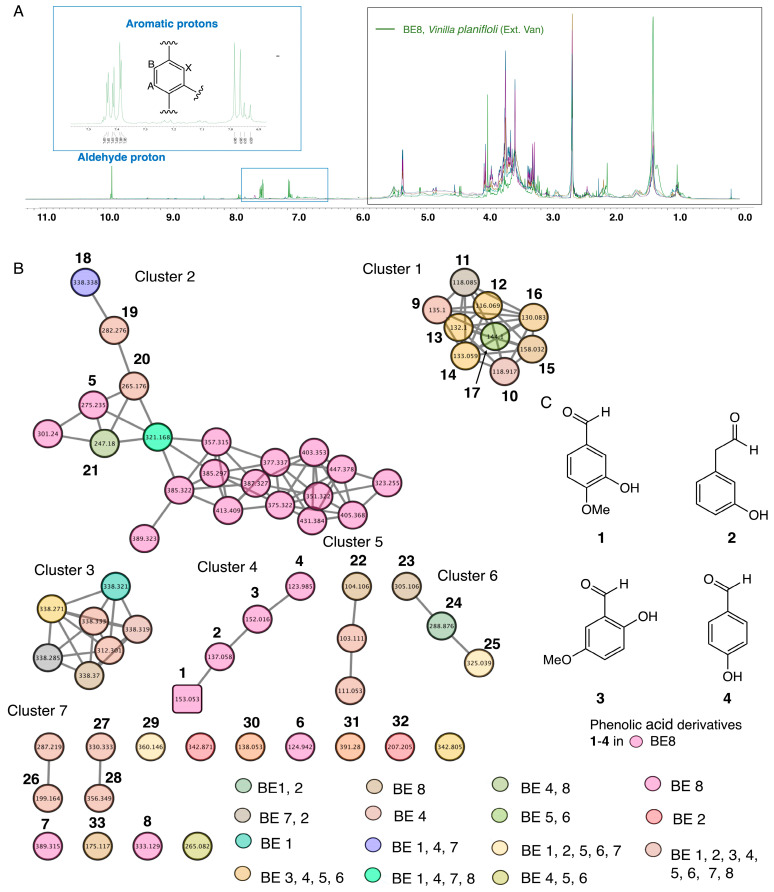
(**A**) Metabolite profiling and fingerprinting of selected bean extracts (BEs: BE1–BE8). Comparison of proton nuclear magnetic resonance (^1^H-NMR) spectra of BE1–8 samples. (**B**) Global natural product social (GNPS) molecular networking (MN) generated from BE1–8 collected from the Hokkaido area. (**C**) Structure of identified phenolic acid derivative metabolites using GNPS.

In this study ^1^H-NMR profiles of selected bean samples were obtained; the ^1^H-NMR spectra are presented in Figure 1A. ^1^H-NMR profiles indicated the presence of a glycoside moiety with a chemical shift between 1 and 6 ppm, suggesting the presence of glycoside metabolites in the selected bean samples (BE1–8). In addition, compared to all measured selected bean samples (BE1–8), ^1^H-NMR spectra of BE8 showed characteristic chemical shifts that indicated peaks of aromatic protons with the ABX system compared to all samples (BE1–7). BE8 showed a specific aromatic chemical shift between 6 and 9 ppm, indicating the presence of phenolic acid derivative metabolites. Metabolite profiling by ^1^H-NMR and fingerprinting of selected BEs (BE1–8) revealed signatures of metabolites related to phenolic aldehydes. NMR measurement of BE8 indicated that the chemical shifts corresponded to vanillin derivative compounds (Figure 1A). Previous reports have suggested that vanilla bean (*Vainilla planifolia*) contains several components, such as phenolic acid derivatives, some of which have vanillin derivatives as one of their major constituents [30,31]. Subsequently, HPLC profiling of the selected bean samples was performed, and additional, prominent peaks were observed in BE8 compared to other extracts, such as BE2, 4, and 5. Similar to the NMR results, HPLC chromatogram-specific peaks were observed for BE8 (Figure 1A, Figures S2 and S3).

#### 2.1.3. LC–MS/MS Analysis and Global Natural Product Social-Aided Dereplication of Constituents from Selected Bean Extracts

GNPS, a global interactive online platform, is one of the leading dereplication platforms for natural product chemistry. GNPS, which includes a mass spectrometry search tool (MASST) coupled with a reference database of food metabolites, is emerging as a powerful resource for understanding the molecular landscape of foods [32,33,34,35]. The metabolomic mass profiles of the eight selected BEs collected from the Hokkaido area were screened using the GNPS based on MS/MS data in the positive ionization mode. Metabolites are represented by nodes in the molecular network, with the chemically related metabolites clustered together. The network was displayed as nodes to reflect the parent ion of each analyzed BE sample (Figure 1B). In this study, tandem mass spectra were obtained from high-resolution mass spectrometers; they can be analyzed and clustered, allowing the creation of molecular networks and annotations of molecules in the database [36,37,38]. The results showed 62 nodes assigned to the bean’s parent ions and 7 constructed clusters (Figure 1B).

The seven clusters were characterized as follows: Cluster 1: Nine parent ions were detected in BE1–8 as compounds **9** and **10**. Compound **11** was detected in BE6 and BE7. Compounds **12**–**14** were detected only in BE7. Compounds **15** and **16** were detected in BE3–6 by using GNPS. Cluster 2: It contained 21 nodes; among them, a parent ion corresponding to **18** was detected in BE1, 4, and 7. Compound **19** was detected in BE1–8, and compound **20** in BE1, 2, and 4. Compound **21** was detected in BE4 and BE8 by using GNPS and unknown parent ions. From the remaining 16 nodes, only 1 was detected in BE7 and identified as **5**; the remaining 15 were unknown. Cluster 3: Seven node isomers were detected in BE2 and 7. Cluster 4: Four nodes were predominantly detected in BE8, corresponding to **1**–**4** metabolites. Cluster 5: Three nodes were constructed. The parent ions was identified as compound **22** and detected in both BE7 and 9. The other two parameters were unknown. Cluster 6: Contained three nodes. The parent ion was identified as compound **23** and detected only in BE7. Compound **24** was detected in BE1 and 2, and compound **25** in BE1, 2, 5, 6, and 7. Cluster 7: This cluster contained 15 nodes; among them, 11 parent ions detected in BE7 were identified as **7**. Compounds **26**–**28** were detected in BE1–8. Compound **29** was detected in the BE1, 2, 5, 6, and 7. Compounds **30** and **31** were detected in BE1–7. Compound **6** was detected in BE8, compound **32** in BE2, and compound **33** in BE6 and 8. The remaining four parent ions are unknown.

Metabolite dereplication revealed that 33 parent ions matched with known metabolites (**1**–**33**) in the GNP library. The GNPS molecular cluster generated from selected BE1–8 identified 33 metabolites and phenolic aldehydes, including vanillin (**1**). LC–LTQ–MS–MS, and MN analyses resulted in the tentative identification of four simple phenolic aldehyde derivatives (**1**–**4**) detected only in BE8, suggesting a metabolic signature for BE8 (Figure 1C). Thus, phenolic metabolite derivatives are considered chemotaxonomic markers of BE8s [25,26]. FooDB is the world’s largest and most comprehensive resource in the chemistry and biology of food constituents. GNPS analysis revealed dereplication of vanillin compound **1**. Compound **1** (*m*/*z* = 153.5321), was submitted to FooDB for confirmation. FooDB discovered that the primary ID of vanillin (**1**) was FDB000838, and the name FooDB corresponded to vanillin (**1**) at CAS 121-33-5. Furthermore, FooDB discovered that vanillin (**1**) is found in foods, such as grains, cereal products, whole grains, cereal products, nuts, legumes, fruits, vegetables, fats, oils (including vegetable oils and olive oils), and beverages (including coffee products, herbal teas, tea products, alcohols, and fermented alcohols such as wine). In this study, the phenolic aldehyde metabolite identified as vanillin was predicted to be a precursor of the derived alkaloid beta-carboline, which contains a C-1 substituted vanillin moiety and can be produced via a one-pot Maillard reaction.

Vanillin, a compound widely used in foods, beverages, cosmetics, and drugs, has demonstrated multifunctional effects, including antimutagenic, anti-angiogenic, anti-colitis, anti-sickling, and anti-analgesic effects. Previous studies have revealed that vanillin exhibits significantly stronger antioxidant activity than ascorbic acid and Trolox in the ORAC assay and OxHL-IA. These findings suggest that the antioxidant activity of vanillin and related phenolic derivatives may be more valuable than previously thought for daily healthcare. Therefore, it was proposed that the antioxidant capacity should be added to the list of the multifunctional effects of vanillin [30,31]. 

### 2.2. Heated Treatment and Acid Modification of Vanilla Bean Extract and Its Dereplication of Metabolites by LC-MS

#### 2.2.1. Chemoselective Derivatization of Vanilla Bean Extract

**Figure 2:**
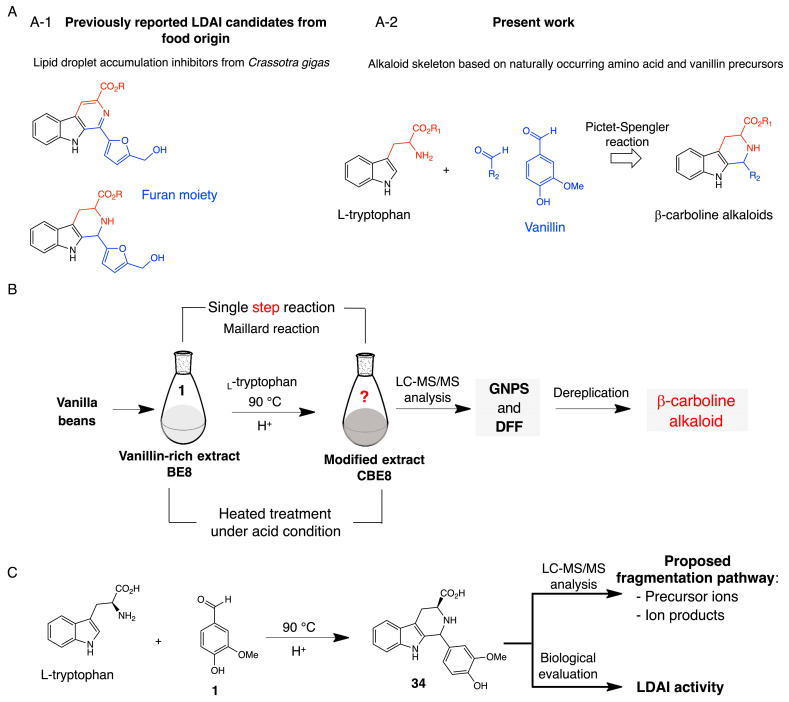
(**A**) (**A-1**) Identified lipid droplet accumulation inhibitors (LDAI) from food origin. (**A-2**) Design strategy for a β-carboline alkaloid using naturally occurring amino acid and phenolic aldehyde precursors. (**B**) Chemo-selective derivatization of vanilla beans (BE8s) and dereplication strategy in the modified bean extract (CBE8). (**C**) Chemical preparation of **34** for the LDAI assay and proposed fragmentation pathway.

L-tryptophan, an aromatic amino acid, was added to BE8 containing the identified vanillin metabolite and heat-treated under acidic conditions. Direct chemical derivatization of BE8 was performed according to a previously described method [15]. The reaction was stopped after 1 h, the mixture was cooled to room temperature and concentrated under vacuum, and a brown solid was obtained. Chemoselective derivatization of BE8 was performed (Figure 2A–C). Therefore, in addition to various chemical changes in the mixture, it was speculated that vanillin and the amino acids in a food extract might readily react under similar conditions to yield β-carboline alkaloids with a vanillin moiety at C-1 in a heated mixture containing vanillin and an excess of L-tryptophan. Maillard, or non-enzymatic browning, occurs among amines and carbonyl constituents, particularly reducing sugars, to produce alkaloidal β-carbolines [11,12,13,14,15]. The β-carboline alkaloids are natural and synthetic indole alkaloids. Some β-carboline alkaloids, including those found in various foods and plants, are widely distributed in nature. A wide range of pharmacological properties has been reported in the literature, including the LDAI activity of flazin containing a pyridine C-ring and its derivative compound containing a piperidine C-ring in OA-loaded HepG2. Flazin and its piperidine C-ring derivative showed low cytotoxicity and significant LDAI activity. These results suggest that β-carboline alkaloids may contribute to the prevention of NAFLD [15,26,27,28]. The GNPS approach (with MN), FoodMASST, DFF, and FooDB were used to investigate metabolite changes in the crude extracts before and after derivatization [17,18,19,20,21,22,32,33]. Inspired by food-derived lipid droplet accumulation inhibitors, a strategy was developed to produce β-carboline alkaloids using naturally occurring amino acids and phenolic aldehyde precursors. Chemical preparation of **34** was achieved by heat treatment under acidic conditions. Chemically prepared compound **34** was used to propose the fragmentation pathway and LDAI test.

#### 2.2.2. Molecular Networking and Detection of β-Carboline **34** in Modified Bean Extract

LC-MS Metabolomics can facilitate a comprehensive view of the changes in a biological system due to the complex linkages between genes and the external environment. The metabolic components of agricultural products and foods are affected by various factors including soil, irrigation, temperature, and altitude. These factors contribute to determining the food phenotype, which is reflected in a specific metabolic fingerprint [29]. High-resolution MS, along with nuclear magnetic resonance (NMR) spectroscopy, is one of the main analytical techniques used to conduct metabolomic experiments. They are powerful tools for investigating the chemical characteristics of plants and foods and provide evidence on the traceability, authenticity, and provenance of agricultural products, as well as their behavioral responses in the context of handling, storage, and processing [30]. Metabolomics has an emerging role in monitoring the influence of different manufacturing procedures on food diversity and security. The metabolomic mass profile of the converted BE8 was investigated using GNPS based on MS data in positive ionization mode.

**Figure 3:**
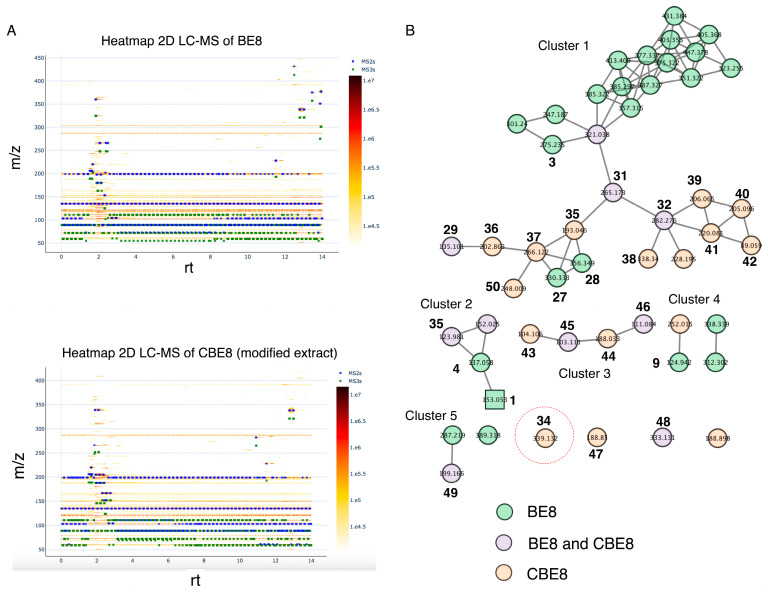
(**A**) A heatmap representation of bean extract 8 (BE8) and modified BE8 was identified by two-dimensional (2D) liquid chromatography/mass spectrometry (LC-MS). (**B**) Global Natural Product Social (GNPS) molecular networking (MN) of modified BE (CBE). Red cercle: Detected β-carboline with a piperidine C-ring and a vanillin moiety at C-1.

The ion parents from BE8 and modified BE8 samples were compared by analyzing the LC-MS data via GNPS platform. The fluctuation of metabolites was visualized by comparing the 2D LC-MS heat map from the GNPS of BE8 and the transformed BE8. A clear chemical change was observed in derivatized BE8 sample, between 12 and 14 min and 300–450 *m*/*z*, a decrease in metabolites was observed, and an increase between 1 and 2 min and 150–300 *m*/*z* compared to BE8. This partially suggests the cleavage of glucoside metabolites to form aglycone metabolites and the appearance of new metabolites, including **34**. The GNPS molecular network generated the data of ion parent detected from the BE initial sample (in green color), BE derivatized sample (purple), and a combination of both extract samples, leading to the identification of 16 molecules (**34**–**50**), including the unknown ion parent predicted to be β-carboline alkaloid **34** (Figure 3). The ion parents are represented by nodes in the molecular network (MN), with chemically related metabolites clustered together. The color of each node indicates the extracts with the corresponding parent ions detected in the MN. Thus, in the network chart, the colored nodes differ from the parent ion detected in the initial BE8 sample and after derivatization (CBE8) (Figure 3). In this study, tandem mass spectra were obtained from high-resolution mass spectrometers using Orbitrap technology, analyzed, and clustered, allowing the creation of molecular networks and annotations of the molecules in the database [31,36]. The results demonstrated that 51 nodes were assigned to the parent ions of the beans, organized as 5 constructed clusters (Figure 3B).

Cluster 1 contains 32 nodes. Among them, 3 parent ions were identified in BE8 as compound **3**, **27**, and **28**, 9 parent ions were identified in the derivatized extract CBE8 (**35**–**42**, **50**), and three compounds **29**, **31**, and **32** were identified in both extracts. Cluster 2 contained four nodes, compounds **1** and **4** were detected in BE8 and compounds **35** and **36** were detected in both BE8 and CBE8. Cluster 3 contained four nodes and parent ions detected in the extract after modification as compounds **43** and **44**, respectively. Compounds **43** and **44** were detected in the CBE8. Cluster 4 contained four nodes; compound **9** was identified in BE8, together with two unknown parent ions, and one additional unknown parent ion in CBE8. Finally, cluster 5 contained seven nodes where compounds **34** and **47** were identified in CBE8 and compounds **48** and **49** were identified in both BE8 and CBE8 samples; the remaining detected parent ions were unknown. Moreover, in cluster 5, among the detected parent ions, 339.1339 was identified as a newly formed β-carboline alkaloid **34** in CBE8, which was absent in BE8 before modification, converting L-tryptophan to β-carboline with a piperidine C-ring and vanillin at the C-1 position (**34**). LC–MS/MS and MN analyses tentatively identified 15 metabolites (**35**–**50**) and unknown compound **34** in the GNPS database.

#### 2.2.3. Structure Characterization and Proposed Fragmentation Pathway of Detected β-Carboline **34**

**Figure 4:**
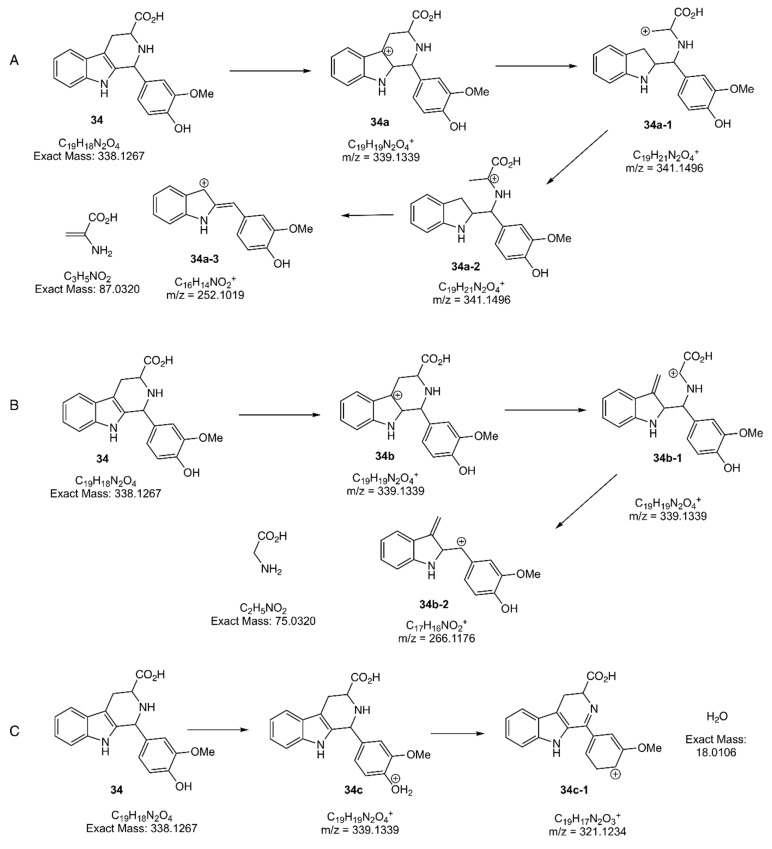
Proposed mass fragmentation pathway of compound **34**. Tandem mass spectrometry (MS/MS) fragmentation shows prominent product ions (**A**) **34a-3**, (**B**) **34b-2**, and (**C**) **34c-1**. π, ionization on π bond; i, inductive cleavage; i, rearrangement; H^+^, protonation; -H_2_O, dehydration.

The detected unknown parent ion 339.1339 (**34**) in the GNPS database was predicted to be β-carboline with a piperidine C-ring and a vanillin moiety at C-1 corresponding to MS^1^: 339.1339 (C_19_H_19_NO_4_^+^). Thus, in this study, the targeted compound **34** was chemically prepared and its structure was confirmed by extensive spectroscopical analysis of the β-carboline alkaloid compound (^1^H-NMR, ^13^C-NMR, and Dept 135, Supporting Information). Then, the fragmentation behavior of **34** with a piperidine C-ring and a vanillin moiety at C-1 was proposed based on the observed MS^2^ fragment values. Compound **34** showed a mass loss for heterocyclic-β-carboline indole derivatives. The losses can be explained by eliminating NH_2_CH_2_COOH, NH_2_(CH_2_) CHCOOH, and H_2_O for the main ion products **34a**, **34b**, and **34c**, respectively. The fragmentation mechanism for the main ion product of **34a-3**, with an abundance of 100%, was initiated by ionization of the π bond at the indole. Next, a cleavage was made within a cation at C-4a, followed by the rearrangement of the cation at C-4, and finally, by the elimination of NH_2_(CH_2_) CHCOOH to obtain an ion on the product of **34a-3** (Figure 4A). The mechanism of fragmentation of the main ion product of **34b-2** (Figure 4B), with an abundance of 30%, was initiated by ionization of the π bond at the indole double bond; successive inductive cleavage (*i*) generated the main ion product of **34b-2** with a cation. The ion product **34c**, with an abundance of 100%, was obtained by protonating the hydroxy group on the phenolic moiety, followed by the elimination of H_2_O, generating an ionic 2-methyl furan moiety at C-1 on **34c-1** (Figure 4C). The proposed mass fragmentation pathway of β-carboline is in agreement with similar compounds reported in the literature [15,34,35].

The fragmentation behaviors of **34** with a piperidine ring having a vanillin moiety at C-1 were proposed based on the observed MS^2^ fragment values (*m*/*z*). (A) Compound **34** exact mass: 338.1339 (C_19_H_18_N_2_O_4_); precursor ion (**34a**) MS^1^: 339.1339 (C_19_H_19_NO_4_^+^); inducted ion (**34a-1**) MS^1^:339.1339 (C_19_H_19_NO_4_^+^); rearranged ion (**34a-2**) MS^1^:339.1339 (C_19_H_19_NO_4_^+^); product ion (**34a-2**) MS^2^: 266.1176 (C_17_H_16_NO_4_^+^); neutral loss:75.0320 (C_3_H_6_NO_2_). (B) Compound **34** exact mass:338.1339 (C_19_H_18_N_2_O_4_); precursor ion (**34b**) MS^1^: 339.1339 (C_19_H_19_NO_4_^+^); inducted ion (**34b-1**) MS^1^: 339.1339 (C_19_H_19_NO_4_^+^); product ion (**34b-2**) MS^2^: 266.1176 (C_17_H_16_NO_4_^+^); neutral loss: 75.0320 (C_2_H_5_NO_2_). (C) Precursor ion (**34c**) MS^1^: 338.1339 (C_19_H_18_N_2_O_4_); product ion (**34c-1**) MS^2^: 252.1019 (C_16_H_14_NO_4_^+^); neutral loss: 18.0320 (C_2_H_5_NO_2_). The fragment ion products, **34a-3**, **34b-2**, and **34c-1**, together with the loss of the NH_2_CH_2_COOH, NH_2_(CH_2_) CHCOOH, moiety, and dehydrated (-H_2_O) parts, revealed β-carboline **34** metabolites with *m*/*z* = 339.1339 (Figure 4C). LC-MS profiling of the methanolic extract was performed using a GNPS-molecular network.

The present study proposes fragmentation pathway for β-carboline alkaloid **34**. Therefore, a β-carboline alkaloid **34** with a piperidine C-ring substituted with a vanillin moiety at C-1 could be detected in the mixture. To detect and identify the compound, specific MS/MS fragmentation criteria for the targeted molecule, diagnostic fragmentation, and a neutral loss filter are required, along with optimized instrument conditions [33].

#### 2.2.4. Identification of **34** in BE8 Using the Specific Tandem Mass Spectrometry Fragmentation

LC–MS/MS and MN analysis tentatively identified 16 metabolites (**34**–**50**), including **34,** predicted to be β-carboline alkaloid with a piperidine C-ring and a vanillin C-1 moiety. A mass fragmentation pathway was proposed for synthetically prepared compound **34,** and MS/MS fragmentation revealed prominent product ions (A) **34a-3**, (B) **34b-2**, and (C) **34c-1**. The ionic products were used to detect β-carboline alkaloid in a mixture of derivatized BE8 via liquid chromatography-MS. Using the result of the proposed fragmentation pathway of **34** (Figure 4), screening of the modified extract led to the identification of the β-carboline alkaloid **34** by the GNPS approach by defining the specific MS/MS fragmentation criteria for the target compound **34**.

LC/MS profiling of modified BE8 was performed using GNPS-molecular networking. Identification of the detected compound **34** in the mixture of modified BE8 was performed by exploring the heatmap and LC-MS/MS analysis with GNPS. The GNPS approach revealed that the MS2 ion product **34b-2** is related to fragmentation B (Figure 4B). The analysis provided strong evidence for the identification of β-carboline alkaloid **34** in the modified crude extract after derivatization of vanilla BE. The parent ion, 339.1339 was identified in cluster 5 as a newly formed β-carboline alkaloid **34** in the modified BE8, which was absent before modification; suggesting the conversion of L-tryptophan to β-carboline with a piperidine C-ring and a vanillin moiety at the C-1 position (**34**). The identification of **34** in the modified extract was confirmed using the complementary DFF approach, as shown in the 2D LC/MS representation plot (Figure S6). This evidence suggests that LC/MS measurement and analysis of the modified extract revealed the presence of the newly formed secondary metabolite **34** and the remaining L-tryptophan in the crude extract of derivatized BE8. It was possible to identify **34** as a newly formed chemical constituent in the modified BE by comparison with the in-house β-carboline alkaloid synthetic standard. Identification of **34** was possible using a complementary DFF approach. Further research on analogous metabolites is required to fully explore the bioavailability of similar molecules in foods with piperidine C-rings substituted with various moieties at C-1.

### 2.3. Cell Viability, Lipid Droplet Accumulation Inhibition Activity, and the Effect of β-Carboline Alkaloid 34 on Lipid Metabolism

Hepatocellular carcinoma (HCC) is the sixth most common cancer worldwide and the third most common cause of cancer-related death. In recent decades, non-viral causes of hepatocellular carcinoma (HCC) have emerged, the most important of which is non-alcoholic fatty liver disease (NAFLD). Intracellular accumulation of lipid droplets (LDs) in the liver is the initial stage of NAFLD. An increasing number of studies have reported an association between excessive intracellular LDA and obesity, diabetes, and other metabolic disorders [15,36,37,38,39,40,41,42,43,44]. Hepatic LDA is thought to be involved in the early stages of NAFLD. NAFLD is the world’s most common chronic liver disease associated with obesity, insulin resistance, type 2 diabetes mellitus, hypertension, dyslipidemia, and metabolic syndrome. Nonalcoholic steatohepatitis (NASH) is a progressive form of NAFLD, that can lead to cirrhosis, hepatocellular carcinoma, and liver failure [36,37,38,39,40,41,42,43,44]. Unfortunately, there are no approved drug therapies for NASH. Therefore, there is an urgent need to establish strategies for the prevention and management of NAFLD/NASH. Agents that can reduce LDA in hepatocytes/livers represent promising candidates for preventing and managing NAFLD/NASH including its HCC and obesity-associated NAFLD [15]. Biologically active natural products, including food-based bioactive alkaloid compounds with LDAI activity in hepatocytes, are potential candidates. Medicinal plants and food are the major sources of alkaloids, including β-carbolines. β-Carbolines, including harman, norharman, flazin, and their derivatives, are present in several plants, fermented and in heat-treated foods [12,13,14,15]. Therefore, there is considerable interest in the relationship between the discovery of bioactive alkaloids and LDAI activity and the prevention of liver diseases, such as NADFL/NASH and related diseases. Cell viability and potential LDAI activity in OA-loaded HepG2 cells were examined to assess the health benefits of alkaloid compound **34**.

#### 2.3.1. Cell Viability: Evaluation of Cytotoxicity and Lipocytotoxicity of β-Carboline Alkaloid **34** in Oleic Acid-Loaded HepG2 Cells

**Figure 5:**
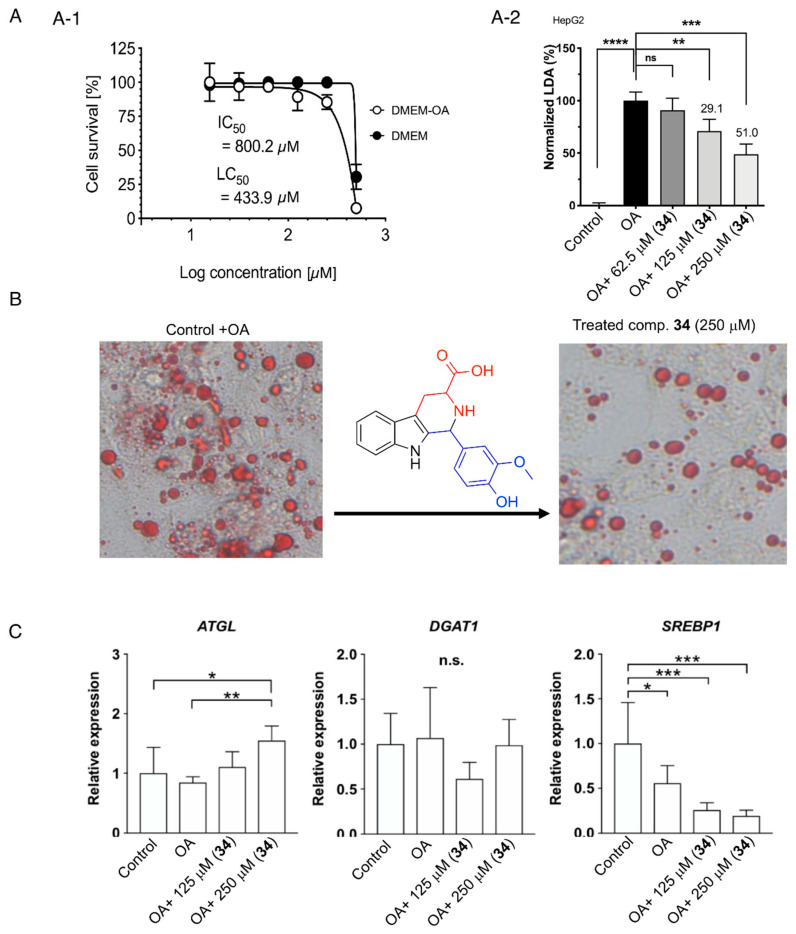
(**A**) (**A-1**) Cell viability of **34** in HepG2 cells. The cytotoxicity (CC_50_) was expressed as the concentration at which 50% of cells died in DMEM without fatty acid (−OA). The lipocytotoxicity (LC_50_) was expressed as the concentration at which 50% of cells died, especially in DMEM loaded with fatty acid (+OA). (**A-2**) LDA inhibition (LDAI) activity of **34** in HepG2 cells. Graph showing the mean values of the LDAI (four replications). **** *p* < 0.0001, *** *p* < 0.001, ** *p* < 0.01 when compared with the untreated control (+OA) group. ns: not significant. (**B**) LDAI activity of compound **34** in HepG2 cells: phase contrast images showing LDs (red); (**C**) Gene expression. All data were normalized to the expression of glyceraldehyde-3-phosphate dehydrogenase (*GAPDH*) and were represented as the relative expression based on that of the control group. *ATGL*: adipose triglyceride lipase; *DGAT1*: diacylglycerol O-acyltransferase 1; *SREBP1*: sterol regulatory element-binding protein 1. ** *p* < 0.01, *** *p* < 0.001, **** *p* < 0.0001 (*n* = 6 in each group). *: statistically significant and n.s.: not significant.

The cytotoxicity and lipocytotoxicity of **34** were evaluated in human HepG2 cells using a CCK-8 assay. The values of the representative species in the mean ± SD (*n* = 6) are given as IC_50_ 800.2 μg/mL for cytotoxicity and LC_50_ 539.0 μg/mL for lipocytotoxicity. The logarithmic plots of cell viability for **34** are shown in Figure 5A. The results showed that compound **34** had no cytotoxicity or lipocytotoxicity in oleic acid-loaded HepG2 cells.

#### 2.3.2. Lipid Droplet Accumulation Inhibition Activity and the Effect of β-Carboline Alkaloid **34** on Lipolysis and Lipogenesis in Oleic Acid-Loaded HepG2 Cells

This study investigated LDAI activity and regulation of lipid metabolism in HepG2 cells that formed LDs upon supplementation with OA (Figure 5A,B). Furthermore, this study evaluated the potential effects of the identified β-carboline alkaloids **34**, derived from modified vanilla BE using heat and acid treatment, on LDA in HepG2 cells. The precursors of these bioactive β-carboline alkaloids were natural, food-occurring aromatic amino acids, L-tryptophan, and vanillin, from the extracts. Food-derived compound **34** inhibited LDA in HepG2 cells treated with OA at 125 μM. In addition, LDs were significantly inhibited by compound **34** in a concentration-dependent manner (Figure 5C). Gene expression analysis was performed to investigate the effects of **34** on lipolysis and lipogenesis (Table S2). Compound **34** was supplemented under LDA conditions in HepG2 cells, and lipolysis- and lipogenesis-related gene expressions were analyzed.

First, ATGL, which plays a critical role in the hydrolysis of triglycerides (TGs), was upregulated upon supplementation with compound **34** under LD formation conditions. Therefore, compound **34** reduced LDA by downregulating ATGL expression. In contrast, the expression of DGAT1, an enzyme involved in TG synthesis, did not change among the groups. Next, the expression of SREBP1, which is related to de novo lipogenesis (DNL) including fatty acid biosynthesis, was investigated. Upon compound **34** supplementation, the expression of these genes was strongly downregulated (Figure 5C). Therefore, compound **34** is associated with the suppression of fatty acid biosynthesis. These results indicated that compound **34** may inhibit LD formation by suppressing fatty acid synthesis and upregulating lipolysis. Detailed lipidomic studies are required for hepatocytes’ free fatty acid (FFA)-loaded cells. Moreover, a systematic investigation of the bioavailability of related metabolites with different skeletons in various natural resources and derivatized crude extracts is required to identify more potent LDAI metabolites for drug development.

## 3. Materials and Methods

### 3.1. Chemicals and Materials

The chemicals and equipment used in this study are described in supporting information, as previously reported [15] (Materials and Methods, Chemicals and instrument).

The following beans materials were used in this study: The eight bean samples used in this study were collected by DDF, SO and NT from the Sapporo market in April 2020 and deposited at the Health Innovation Center of the Faculty of Health Sciences at Hokkaido University. They were listed as [Code, Scientific name (abbreviation)]: BE1, *Glycine max*; BE2, *Glycine max*; BE3, *Vigna angularis*; BE4, *Phaseolus vulgaris*; BE5, *Phaseolus coccineus*; BE6, *Phaseolus coccineus*; BE7, *Vicia faba*; BE8, *Vinilla planiflolia*.

### 3.2. Determination of Antioxidant Activity and TPC

The TPC of the eight selected BEs (BE1–BE2) was determined using a modified Folin–Ciocalteu method [45]. Briefly, 100 μL of crude extract (1 mg/mL) was mixed thoroughly with 0.5 mL of 10% phenol reagent, followed by the addition of 0.4 mL of 7.5% (*w*/*v*) sodium carbonate within 3–8 min. The mixture was allowed to stand for a further 60 min in the dark, and absorbance was measured at 765 nm. The TPC was calculated from the calibration curve of the gallic acid standard. The results are expressed as milligrams of gallic acid equivalent per gram of dry weight. The antioxidant activity of the extract was determined by the 1,1-diphenyl-2-picryl-hydrazyl (DPPH) assay, as described previously, with some modifications [45]. Briefly, 20 μL of each extract (100–500 μg/mL) was mixed with 80 μL PBS in a 96-well plate. Next, 0.1 mL DPPH solution was added. Samples were then incubated in the dark at room temperature for 30 min. The absorbance of the mixture was measured at 745 nm using Multiskan FC (Thermo Fisher Scientific K. K., Tokyo, Japan). Ascorbic acid was used as the positive control.

### 3.3. Metabolite Profiling of Vanilla Bean Extracts 

The bean samples selected were BE1, BE2, BE3, BE4, BE5, BE6, BE7, and BE8. The samples were extracted using MeOH for three cycles at room temperature. The extracts were concentrated under reduced pressure to obtain a methanolic extract. Proton nuclear magnetic resonance (^1^H-NMR) measurements and liquid chromatography/mass spectrometry (LC-MS) of all BE extracts (BE1–BE8) were performed.

#### 3.3.1. Nuclear Magnetic Resonance Profiling of Vanilla Bean Extract 

^1^H-NMR measurements of all the BEs (BE1–BE8) were performed using a JEOL ECX400 Delta spectrometer with TMS as an internal standard; chemical shifts were expressed as *δ* values.

#### 3.3.2. Liquid Chromatography/Mass Spectrometry Profiling of Vanilla Bean Extract

A. LC-MS instrument conditions and processing

In this study, 3D LC-MS and DFF were used. Samples of methanol BEs were separated using an Atlantis T3 C18 column (2.1 × 150 mm, 3 μm, 155 Waters, Milford, MA, USA). A representation of the workflow is shown in the Supporting Information (Figure S3) at a flow rate of 200 μL/min. The LC-MS instrument conditions used in this study have been previously described [15] in Supporting Information: Materials and Methods, Chemicals and instruments. MS/MS spectra were obtained, and the raw data were processed using Xcalibur 2.2 (Thermo Fisher Scientific Inc., San Jose, CA, USA), as described previously. The workflow used here follows a general pipeline for untargeted LC-MS (or LC-MS/MS) data preprocessing, where the main goal was to turn the highly complex LC-MS raw data into a list of features and the corresponding signal intensity, detected across the analyzed BE sample. The feature lists were then exported for further downstream analysis (e.g., 3D LC-MS, DDF, identification, and search against spectral libraries).

B. Molecular network of BE (BE1–BE8) samples

A classical molecular networking (MN) workflow using the GNPS platform was performed, and a molecular network of all selected BE (BE1–BE8) was created using the online workflow on the GNPS website. The data were filtered by removing all MS/MS fragment ions within the precursor ±17 Da. Next, MS/MS spectra were window-filtered by choosing the top six fragment ions in the ±50 Da window throughout the spectrum. The precursor ion mass tolerance was set to 2.0 Da, and an MS/MS fragment ion tolerance of 0.9 Da. Then, a network was created where edges were filtered to have a cosine score above 0.5 and more than six matched peaks. Furthermore, the edges between two nodes were retained in the network only if each node appeared in the top 10 most similar nodes. Finally, the maximum size of a molecular family was set to 100, and the lowest-scoring edges were removed from molecular families until the molecular family size was below this threshold. The spectra in the network were then searched against the GNPS spectral libraries.

C. Compounds dereplication using LC-MS

A molecular network was created using the GNPS online workflow. The spectra in the network were then searched against the GNPS spectral libraries, FooDBs, and published data. Metabolite dereplication revealed that 33 parent ions matched with previously reported metabolites (**1**–**33**) in the GNP library. The library spectra were filtered in the same manner as for the input data. All matches between the network and library spectra were required to score above 0.7 and at least six matched peaks. Compounds: vanillin (**1**), phenylacetic acid, 2-hydroxy (**2**), benzaldehyde, 2-hydroxy-4-methoxy (**3**), 4-hydroxybenzaldehyde (**4**), cembrene (**5**), benzaldehyde, 2-hydroxy (**6**), _L_-Asparagine (**7**), phenylacetic acid (**9**), methylmalonic acid (**10**), _L_-Homoserine (**11**), _D_-ornithine (**12**), MFCD00004183 (**13**), N-acetylproline (**15**), aminoadipate (**17**), 13-Docosenamide, (Z) (**18**), 2,6-Diaminohexanoic acid (**16**), 9-Octadecenamide, (Z) (**19**), 1-Oleoyl-2-acetyl-sn-glycerol from NIST14 (**20**), _L_-(-)-Serine (**22AA**), NCGC00380682-01_C13H18O8_(**23**), 4H-Pyran-4-one, 3-(beta-_D_-glucopyranosyloxy)-2-methyl- (**24**), Lactulose (**25**), N-Acetylhistidine (**26**), 1-Hexadecanoyl-sn-glycerol (**27**), bullatine G (**28**), urocanate (**30**), sn-Glycerol 3-phosphate (**31**), and _L_-Arginine (**33**). The DFF module has been implemented into the open-source, platform, and is available by downloading MZmine 2.38 or newer releases. DFF allows users to efficiently screen DDA datasets for MS/MS spectra that contain product ion(s) and/or neutral loss(es), which are diagnostic for all classes of compounds. A limitation of DFF is that the characteristic product ions and/or neutral losses for a class of compounds must be defined by the analyst. Thus, we first synthesized compound **34**, and then explored the proposed fragmentation pathway to find the ion products and/or neutral losses for use as diagnostic product ions (*m*/*z*) and diagnostic neutral loss value (Da) in DFF. DFF was performed using a high-resolution mass spectrometer for the target class of the analytes. 

#### 3.3.3. Chemo-Selective Derivatization of Vanilla Bean Extracts under Acidic Conditions and LC-MS/MS Analysis of the Modified Vanilla Bean Extract

L-tryptophan, an aromatic amino acid containing the identified vanillin metabolite and heat-treated under acidic conditions, was added to BE8. LC-MS experiments were performed as previously described [15]. Briefly, the molecular network was created using the GNPS online workflow [46,47]. The spectra in the network were then searched against GNPS spectral libraries, published data, and in-house standard molecules. β-carboline alkaloid **34** was identified in the modified extract using the DFF and GNPS approaches. LC-MS measurements of converted vanillin BE and standard **34** were performed using a Shimadzu Prominence HPLC system (Shimadzu Corp., Kyoto, Japan) coupled to an LTQ Orbitrap mass spectrometer (Thermo Fisher Scientific Inc., San Jose, CA, USA) with an ESI source.

The classical MN workflow using the GNPS platform was performed as described in Section 3.3.2.B. Metabolite dereplication revealed that 33 parent ions matched known metabolites (**34**–**51**) in the GNP library. The parent ions from liquid chromatography with tandem mass spectrometry (LC-MS/MS) analysis of the modified extracts were dereplicated as follows: β-carboline alkaloids compound (**34**), _L_-5-hydroxy-tryptophan (**35**), _L_-Kynurenine (**36**), hypnophilin (**37**), L-tryptophan (**38**), isocitrate| (**39**), 13a-Hydroxylupanin (**40**), MoNA:2488524 2 (**41**), ethylene Thiourea (**42**), N-Hydroxytetraadecanoylleucine (**43**), putrescine (**44**), 13-Docosenamide (**45**), 1-Hexadecanoyl-sn-glycerol (**46**), abrine (**47**), _DL_-Indole-3-lactic acid (**48**), MoNA:2488004 (**49**), and N-Acetylhistidine (**50**). 

The LC-MS instrument conditions used to analyze the modified vanilla bean extract were the same as those described in Section 2.3.2. The HREIMS feature detection was performed using MZmine 2 (http://mzmine.github.io, accessed on 21 September 2022). The retention time (RT) and MS values of metabolites identified in BE8 were compared to those of the synthesized standard **34**. Thermo Fisher Scientific Xcalibur 2.2. Software was used to analyze results and identify metabolite. Before LC-MS/MS analysis of the selected bean extracts, the raw data files were converted to mzXML format using ProteoWizard’s msConvert. In this study, non-targeted liquid chromatography (LC)-MS/MS datasets and DFF for **34** analyses were prepared. DFF was performed using a high-resolution mass spectrometer. The raw data import option was selected under the raw data methods drop-down menu of MZmine 2. The data files of BE8 to be analyzed were then chosen and imported into MZmine 2. For **34** analyses, the following settings were used within the DFF module: 1. Retention time: Input in the range of 0.00 to 14.00 min; 2. Precursor *m*/*z*: Input *m*/*z* range of 50.00 to 500.00; 3. *m*/*z* tolerance: Apply *m*/*z* tolerance of 0.01 *m*/*z* or 3.0 ppm; 4. Diagnostic product ions (*m*/*z*): input *m*/*z* of 266.1176, 252.1059 as the diagnostic product ions; 5. Diagnostic neutral loss value (Da): Input 75.0320 to define that no diagnostic neutral losses are being used; 6. Minimum diagnostic ion intensity (% base peak): Use 15.00 as the minimum intensity threshold; and 7. Thus, DFF analysis was performed and a DFF plot (Figure S6) was obtained by completing the above steps. 

#### 3.3.4. Preparation Procedure of Identified β-Carboline Alkaloid **34** Using Acidic Conversion

Compound **34**, a newly formed and identified β-carboline alkaloid with a piperidine C-ring and vanillin moiety at C-1, was designed and chemically synthesized. The compound was prepared by dehydration, condensing l-tryptophan and vanillin at the C-1 position. The reaction procedure is illustrated in Figure S1. Vanillin (704.4 mg; 4.62 mmol) was added to L-tryptophan (600 mg; 2.94 mmol) in acetic acid (30 mL). The total solution was observed, and the mixture was refluxed (90 °C). After 3 h, low MS indicated presence of **34**, and the reaction was completed. The mixture was then cooled to room temperature and concentrated to dryness. A silica gel chromatography column was used to purify the yellow solid to yield **34** (63.1 mg). The synthesis method was performed as reported by Chen and al. [48]. Next, an extensive spectroscopic analysis was performed to determine the structure (Supplementary Information, Figure S1). The predicted β-carboline alkaloid **34** was prepared and used to propose the fragmentation pathway and as a standard for defining specific MS/MS fragmentation criteria for diagnostic fragmentation filter analysis and evaluation of its LDAI capacity.

### 3.4. Evaluation of Cell Viability 

Cytotoxicity and lipocytotoxicity assays were performed according to the manufacturer’s protocol using the Cell Counting Kit-8 (CCK-8; Dojindo Molecular Technologies, Rockville, MD, USA), as previously indicated [15] and are described in detail in the additional Supplemental Information. HepG2 cells were purchased from RIKEN BRC cell bank (Ibaraki, Japan).

### 3.5. Lipid Metabolism-Related Gene Expression

Gene expression analysis was performed using real-time PCR to investigate how supplementation of compound **34** during LDA in HepG2 cells affects lipolysis and lipogenesis. After another 24 h, RNA extraction and complementary DNA synthesis were performed according to the manufacturer’s instructions as previously reported [15,49,50] and detailed in the Supplementary Information (Materials and Methods).

### 3.6. Lipid Droplet Accumulation Inhibition Assay

LDAI activity was determined using an Oil Red O assay with 24-well plates (*n* = 4 per treatment) as previously reported [15]. LD staining assay. Staining was performed with modifications, as previously reported [15], and details are reported in the Supplementary Information (Materials and Methods).

## 4. Conclusions

In summary, an antioxidant extract of *V. planifolia* was studied after chemoselective derivatization to diversify the chemical constituents of the extract, monitor metabolite changes using a metabolomic approach, identify predicted derived alkaloid compounds, and evaluate its LDAI activity. Among the eight selected beans, NMR fingerprinting revealed a characteristic signature of phenolic compounds in BE8. Molecular networking using LC-MS/MS analysis of *V. planifolia* through chemoselective derivatization revealed **34**–**50** compounds including a β–carboline alkaloid with a piperidine C-ring and vanillin moiety at C-1 (**34**) in a mixture of modified BE8. The ion products from fragmentation pathway of **34** were reported. Compound **34** exhibited significant LDAI activity in HepG2 cells treated with OA. In addition, compound **34** attenuated LDA by activating lipolysis and suppressing lipogenesis. This study is the first to identify a newly generated alkaloid from modified *V. planifolia* with LDAI capacity in HepG2 cells. This finding suggests that β–carboline alkaloids represent potential bioactive LDAI candidates that might be useful in preventing and managing non-alcoholic fatty liver disease and associated diseases. The study indicates that crude plant extracts, following chemoselective derivatization, may contain bioactive compounds that could be beneficial in preventing hepatosteatosis and serving as a lead compound for drug development.

## Figures and Tables

**Table 1:** Total polyphenol content of BE1–BE8 and DPPH radical scavenging activity of BE1–BE8 as a Trolox equivalent.

Bean Extracts (BEs)	Total Polyphenol Content (μg/mg)	nmolTE/mg Bean Extracts	Bean Extracts (BEs)	Total Polyphenol Content (μg/mg)	nmolTE/mg Bean Extracts
BE1	7.80	4.26	BE5	59.7	0.85
BE2	11.8	3.40	BE6	5.40	5.00
BE3	41.5	5.96	BE7	12.7	7.38
BE4	40.3	3.69	BE8	60.8	7.60

## Data Availability

Data are contained within the article and Supplementary Materials.

## Supplementary Materials

The following supporting information can be downloaded at https://www.mdpi.com/article/10.3390/molecules28248024/s1. Materials and Methods: 1. Chemicals and instruments; 2. Liquid chromatography/mass spectrometry profiling of vanilla bean extract; 3. Lipid metabolism-related gene expression; 4. Lipid droplet accumulation inhibition assay; 5. Statistical analysis; 6. Complementary metabolite identification via DFF and, ^1^H-NMR analyses of selected bean extracts; Identification of **34** using complementary LC-MS/MS diagnostic fragmentation filtering approach; Figure S1: ^1^H NMR, ^1^H-^1^H COSY, ^13^C NMR and Dept 135 spectrum of b-carboline alkaloid (**34**) in CDCl_3_; Figure S2: HPLC profiling of selected bean extracts: The comparison of BE2, BE4, BE5 and BE8 spectra; Figure S3: A Schema of the general data processing workflow of LC-MS data; Figure S4: LC-MS profiling of bioactive bean extracts. (A) Diagnostic Fragmentation Filtering (DFF) plot for metabolites analysis. (B) 3D visualization of MS data; Figure S5: (A) Three-dimensional (3D) liquid chromatography/mass spectrometry (LC-MS) comparison of bean extract (BE) 8 and vanillin. (B) Diagnostic fragmentation filtering (DFF) plot of the vanillin in BE8; Figure S6: Diagnostic fragmentation filtering (DFF) identification of β-carboline alkaloid 34: DFF analysis of 34 in modified vanilla BE -8 mixture after 30 min and 1 h of treatment; Table S1: LC-MS profiling using MZmine of bioactive bean extracts BE2, BE4, BE5, and BE8: Retention time (RT, min), MS1, MS2 and MS3 (*m*/*z*); Table S2: List of primers. Available from authors, GNPS data for MN: Global Natural Product Social (GNPS) molecular networking (MN) generated from BE1–8 collected from the Hokkaido area and Global Natural Product Social (GNPS) molecular networking (MN) of modified BE (CBE). Ref. [51] is cited in the supplementary materials
