# Supplementary material for: Identification of a β-Carboline Alkaloid from Chemoselectively Derived Vanilla Bean Extract and Its Prevention of Lipid Droplet Accumulation in Human Hepatocytes (HepG2)
Source: Molecules. 2023 Dec 9;28(24):8024. doi: 10.3390/molecules28248024 (PMC10745329; doi:10.3390/molecules28248024)
Supplement: Supplementary file 1 [file molecules-28-08024-s001.zip › molecules-2633474-supplementary.pdf]

Identification of a β-Carboline Alkaloid from Chemoselectively Derived Vanilla Bean Extract and Its Prevention of Lipid Droplet Accumulation in Human Hepatocytes (HepG2)

Dya Fita Dibwe [1], Nire Takeishi [2], Saki Oba [2], Akiko Sakurai [2], Toshihiro Sakurai [1], Takayuki Tsukui [3], Hitoshi Chiba [3] and Shu-Ping Hui [1,*]

[1] Faculty of Health Sciences, Hokkaido University, Kita-12, Nishi-5, Kita-Ku, Sapporo 060-0812, Japan; dibwedf@hs.hokudai.ac.jp (D.F.D.); sakura@hs.hokudai.ac.jp (T.S.)
[2] Graduate School of Health Sciences, Hokkaido University, Kita-12, Nishi-5, Kita-Ku, Sapporo 060-0812, Japan; n10461991724nasukakstg@docomo.ne.jp (N.T.); saki.oba.h@gmail.com (S.O.); sakurai.akiko.p6@elms.hokudai.ac.jp (A.S.)
[3] Department of Nutrition, Sapporo University of Health Sciences, Nakanuma Nishi-4-3-1-15, Higashi-Ku, Sapporo 007-0894, Japan; tsukui@sapporo-hokeniryou-u.ac.jp (T.T.); chiba-h@sapporo-hokeniryou-u.ac.jp (H.C.)
* Correspondence: keino@hs.hokudai.ac.jp; Tel./Fax: +81-11-706-3693

Table of contents: **Supporting Information**

Materials and Methods P4

1. Chemicals and instruments P4

2. Liquid chromatography/mass spectrometry profiling of vanilla bean extract P5

3. Lipid metabolism-related gene expression P5

4. Lipid droplet accumulation inhibition assay P6

5. Statistical analysis P6

6. Complementary metabolite identification via DFF and, ^{1}H-NMR analyses of selected bean extracts P6

7. Identification of **34** using complementary LC-MS/MS diagnostic fragmentation filtering approach P7

Figure S1: ^{1}H NMR, ^{1}H-^{1}H COSY, ^{13}C NMR, and Dept 135 spectrum of β-carboline alkaloid (**34**) in $CDCl_3$ P9

Figure S2: HPLC profiling of selected bean extracts: The comparison of BE2, BE4, BE5, and BE8 spectra P10

Figure S3: A schema of the general data processing workflow of LC-MS data P11

Table S1: LC-MS profiling using MZmine of bioactive bean extracts BE2, BE4, BE5, and BE8: Retention time (RT, min), MS^1, MS^2, and MS^3 (*m/z*) P12

Figure S4: LC-MS profiling of bioactive bean extracts. (A) Diagnostic Fragmentation Filtering (DFF) plot for metabolites analysis. (B) 3D visualization of MS data P13

Figure S5. (A) Three-dimensional (3D) liquid chromatography/mass spectrometry (LC-MS) comparison of bean extract 8 (BE8) and vanillin. (B) Diagnostic fragmentation filtering (DFF) plot of the vanillin in BE8. P14

Figure S6. Diagnostic fragmentation filtering (DFF) identification of β-carboline alkaloid **34**: DFF analysis of **34** in modified vanilla BE8 mixture after 30 min and 1 h of treatment P15

Table S2: List of primers P16

1. Materials and Methods

Chemicals and instruments

General Experimental Procedures: Nuclear magnetic resonance (NMR) spectra were recorded using a JEOL ECX400 Delta spectrometer with TMS as an internal standard; chemical shifts were expressed as *δ* values. An LTQ Orbitrap XL mass spectrometer (Thermo Fisher Scientific Inc., San Jose, CA, USA) was used for high-resolution electrospray ionization mass spectrometry (HR-ESI-MS) measurements. Low-resolution electrospray ionization mass spectrometry (LR-ESI-MS) spectra were recorded using an LXQ spectrometer (Thermo Fisher Scientific Inc., San Jose, CA, USA). Analytical thin-layer chromatography (TLC) was performed on pre-coated silica gel $60F_{254}$ and $RP\text{-}18F_{254}$ plates (0.25 or 0.50 mm, Merck KGaA, Darmstadt, Germany). Preparative TLC was performed on pre-coated silica gel 70 PF_{254} plates (0.75 mm thickness; Wako Pure Chemical Industries, Ltd., Japan). Semi-preparative high-performance liquid chromatography (HPLC) separations were performed on a Shimadzu quaternary LC VL instrument with a UG 120 A column (250 × 10 mm; 5 μm I.D.). Methanol was purchased from Wako. High-glucose Dulbecco’s Modified Eagle Medium (DMEM), Dulbecco’s phosphate-buffered saline (DPBS), trypsin EDTA, fetal bovine serum (FBS), and penicillin-streptomycin (100 U/mL) were purchased from Gibco (Life Technologies, Carlsbad, CA, USA). Other materials used for cell culture were purchased from Corning (NY, USA). TLC silica gel 60G F_{254} glass plates (20 × 20 cm) were obtained from Merck (Tokyo, Japan), and spots were visualized by spraying with 5% ethanolic H_2SO_4. ^{1}H, ^{13}C NMR, Distortionless Enhancement by Polarization Transfer (DEPT), and two-dimensional (2D) NMR spectra were acquired using a 400 MHz JNM-ECX400P spectrometer (JOEL, Japan). The spectra were processed using JOEL software, and the chemical shift (*δ*) values were expressed in ppm. OA was purchased from Cayman Chemical (Ann Arbor, MI, USA), and absorbance was measured using ARVO-MX (Perkin Elmer, Waltham, MA, USA) as previously reported (15)

2. *Liquid chromatography/mass spectrometry profiling of vanilla bean extract*

LC-MS instrument conditions

Samples of methanol BEs were separated using an Atlantis T3 C18 column (2.1×150 mm, 3 μm, 155 Waters, Milford, MA, USA) at a flow rate of 200 μL/min. LC gradient elution was performed using a mobile phase of 10 mM ammonium acetate solution, isopropanol, and methanol. The measurements were carried out in positive mode; the voltage of the MS capillary was 4.04 kV, flow rate of the sheath gas (nitrogen) was 50 psi, and auxiliary gas (nitrogen) was 20 psi. The high-resolution MS data were obtained in a scan range of m/z = 150–1100. MS/MS spectra were obtained by data-dependent acquisition using collision-induced dissociation (CID) in ion-trap mode for low-resolution masses. The raw data were processed using Xcalibur 2.2 (Thermo Fisher Scientific Inc., San Jose, CA, USA) as previously described (15).

3. *Lipid metabolism-related gene expression.*

Gene expression analysis was performed using real-time PCR to investigate how the supplementation of compound **34** during LDA in HepG2 cells affects lipolysis and lipogenesis. HepG2 cells (2.0×10^5/well) were seeded into 24-well plates (n = 6 per treatment). After 24 h, the cells were treated with PBS: 0.25 mM PBS for the OA-only group, 0.25 mM PBS for the OA plus group, and 125 and 250 μM PBS for the compound **34** group. After another 24 h, RNA extraction and complementary DNA synthesis were performed according to the manufacturer's instructions and as indicated previously. (15,54,55). The sequences of the primers used are shown in Table S1: adipose triglyceride lipase (*ATGL*), diacylglycerol O-acyltransferase 1 (*DGAT1*), sterol regulatory element-binding protein 1 (*SREBP1*), and glyceraldehyde-3-phosphate dehydrogenase (*GAPDH*). PCR was performed using a CFX 96 Real-Time PCR Detection System (Bio-Rad Laboratories Inc., Hercules, CA, USA) under the following conditions: 95 °C for 30 s, followed by 40 cycles at 95 °C for 5 s, 60 °C for 5 s,

and 72 °C for 10 s. All gene expression levels were analyzed using the ΔΔCt method and normalized to GAPDH. Data were represented as a relative expression based on the control group.

4. *Lipid droplet accumulation inhibition assay*

LDAI activity was determined using an Oil Red O assay with 24-well plates (n = 4 per treatment) based on a First, the staining of LDs in cultured hepatocytes was performed according to the manufacturer's instructions. Next, HepG2 cells (1.5×10^4/well) were supplemented with 10% FBS, cultured, seeded into 35 mm dishes, and treated with the tested samples after 24 h. Oil Red O, a fat-soluble dye, is widely used for staining neutral lipids in LDs, as previously described (12). Next, quantification of LD inhibition was assessed for test compound **34** by comparing it to the untreated control group (+OA) and normalizing the LDA absorbance values (%) as previously reported (15). LD staining assay. Staining was performed as previously described with modifications (15).

5. *Statistical analysis*

For gene expression analysis, mean values were compared using a one-way analysis of variance (ANOVA), followed by Tukey's multiple comparison test. Statistical analyses were performed using GraphPad Prism 8.0e (GraphPad Software Inc., La Jolla, CA, USA). Statistical significance was set at $p < 0.05$.

6. *Complementary metabolite identification via DFF and, ^{1}H-NMR analyses of selected bean extracts*

Natural products are usually synthesized as mixtures of structurally similar compounds instead of as a single compound. Because of their shared structural characteristics, numerous compounds of the same class are subject to similar MS/MS fragmentation and exhibit several identical product ions and/or neutral losses. The objective of DFF is to accurately detect all compounds of a specific class in a complex extract by filtering out non-targeted LC-MS/MS datasets for MS/MS spectra

that contain class-specific product ions and/or neutral losses (13, 27). The LC/MS profiling of the methanolic extract was presented as a 3D LC/MS plot with retention time and MS values (Figure S5-A). The LC/MS measurement and analysis of the vanilla BE revealed phenolic acid derivative metabolites (**1**).

It was possible to identify the phenolic derivative metabolites as major chemical constituents of the extract compared to the in-house standards. DFF is a straightforward and rapid strategy for detecting entire classes of compounds in a mixture of natural food products. DFF is especially relevant for the dereplication and discovery of natural product compounds. DFF was used for screening metabolites from LC-MS/MS datasets of BE8. The complementary results are 3D images and their DFF. The identified vanillin was further detected in the 3D LC-MS comparison of BE8 and vanillin standard and by using the DFF approach, as shown in the graph with the characteristic ion products and precursors from the LC-MS/MS analysis. As shown in the 3D plot in Figure 2A, vanillin (**1**) was found in the extract based on the retention time and MS/MS spectra of the authentic standard. The fragmentation behaviors of **1** were used for DFF analysis (Figure S5-B). The DFF results supported the GNPS and FooDB findings on this BE8 extract. Since vanillin has been previously reported to be effective in treating obesity, it would be interesting to investigate the effect of BE8 extract on NAFLD. Therefore, the LDAI activity of BE8 extract in HepG2 cells needs further investigation.

***7.** Identification of **34** using complementary* LC-MS/MS *diagnostic fragmentation filtering approach*

Natural products are usually synthesized as mixtures of structurally similar compounds instead of as a single compound. Because of their shared structural characteristics, numerous compounds of the same class are subject to similar MS/MS fragmentation and exhibit several identical product ions and/or neutral losses. The objective of DFF is to accurately detect all compounds of a specific class in a complex extract by filtering out non-targeted LC-MS/MS data sets for MS/MS spectra

that contain class-specific product ions and/or neutral losses (15, 32,33). DFF allows for the further discovery of all related natural products in a complex sample (13, 25, 27), but the main limitation of DFF approach is that the analyst must first define which product ions and/or neutral losses are specific to the targeted natural product class. Thus, in this study additionally to the identification of **34** by GNPS-MN analysis, DFF analysis of BE8 was performed as a complementary analysis for the identification of **34**. The proposed fragmentation results from synthetically prepared **34** was used for the DFF analysis. The LC–MS/MS analysis also resulted in the identification of **34** from the modified BE8. This result supported the identification of compound **34** by the GNPS-NM. Compound **34** was identified for the first time from the converted BE (*m*/*z* = 339.1339). The fragmentation pathway (Figure 4-A–C) demonstrates the possible fragments that defined the fragmentation characteristics of compound **34**. This fragmentation can be used for the analysis of the modified BE after heat treatment and acid modification.

The identification of **34** in the modified extract was also confirmed by using the complementary DFF approach, as shown in the 2D LC/MS representation plot (Figures 6). This evidence suggested that the LC/MS measurement and analysis of the modified extract revealed the presence of newly formed β-carboline alkaloid secondary metabolite **34** and the remaining L-tryptophan. It was possible to identify **34** as a newly formed chemical constituent in the modified BE by its comparison with the in-house β-carboline alkaloid synthetic standard. The identification of **34** was possible by using a complementary diagnostic fragmentation filtering approach (Figure S6). In Figure 6, the red dotted line represents the diagnostic product ions required to be considered a putative metabolite **34**. The blue dotted line represents the $[M+H]^+$ ions that are precursors of the β-carboline alkaloid **34**. Further research on analogous metabolites is required to fully explore the bioavailability of similar molecules in foods with a piperidine C-ring substituted with various moieties at C-1.

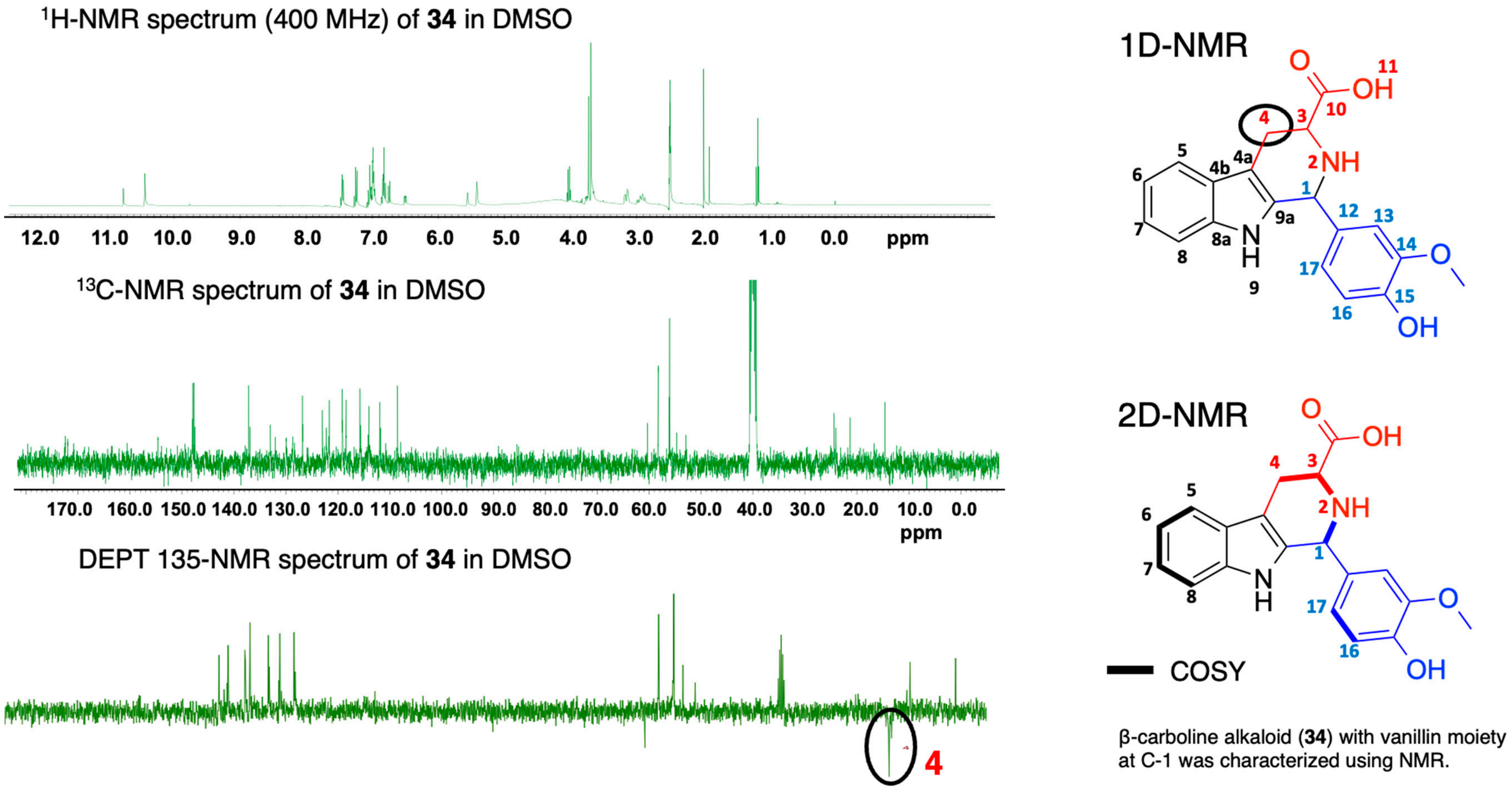

Figure S1. ^{1}H NMR, ^{1}H-^{1}H COSY, ^{13}C NMR, and Dept 135 spectrum of β-carboline alkaloid (**34**) in $CDCl_3$.

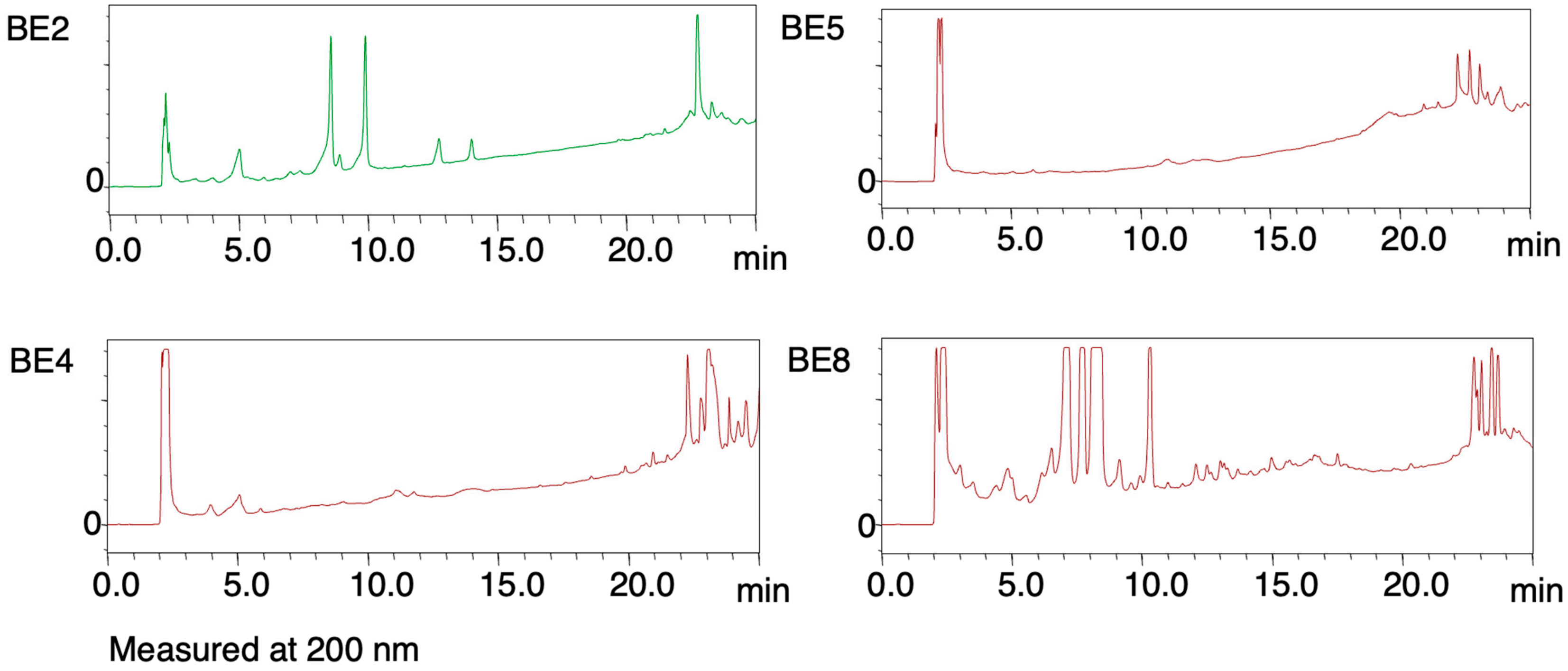

Figure S2. HPLC profiling of selected bean extracts: The comparison of BE2, BE4, BE5, and BE8 spectra.

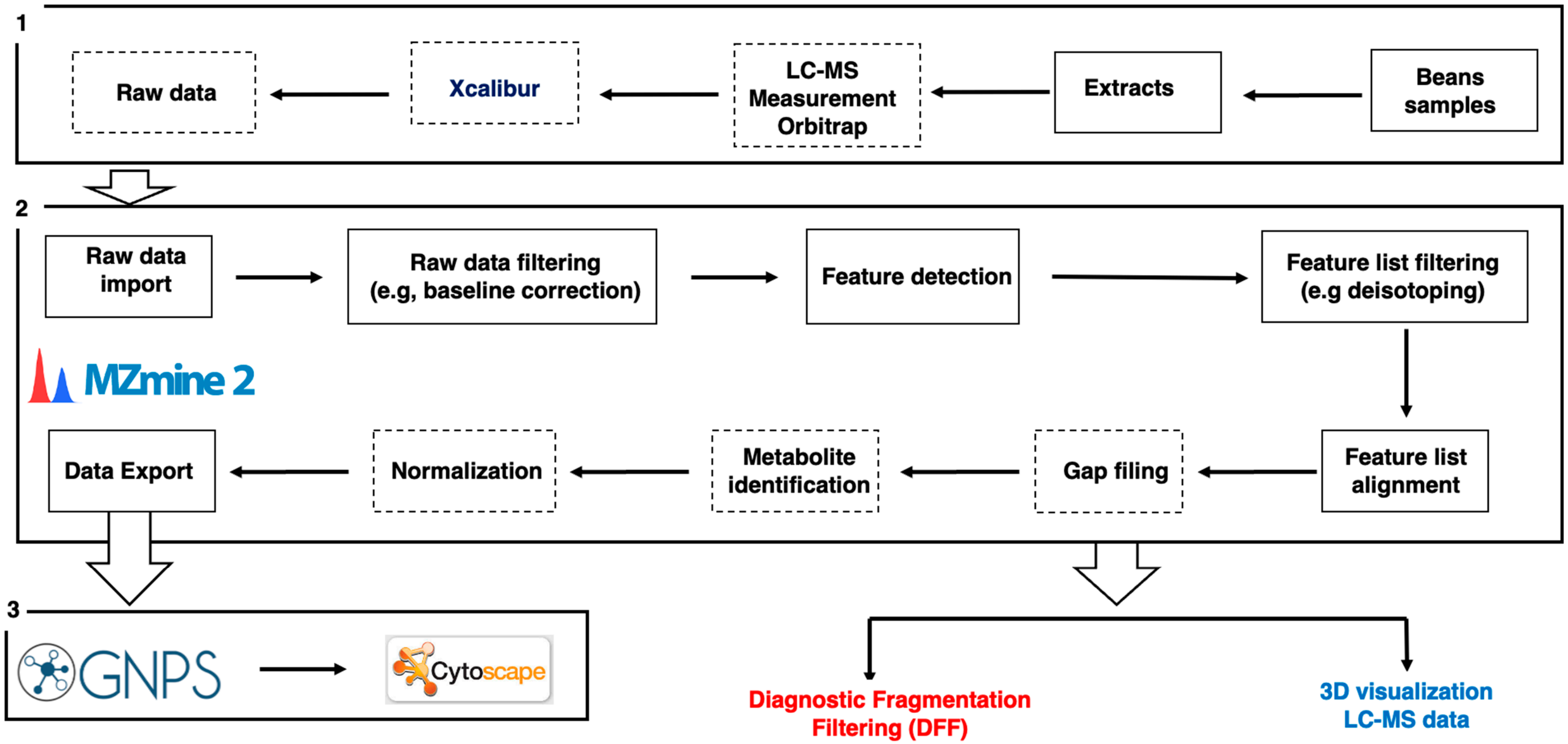

Figure S3. A schema of the general data processing workflow of LC-MS data.

	BE2 (Sample 2)			
	RT	MS1(m/z)	MS2(m/z)	MS3(m/z)
Peak 1	13.8808567	61.063961	61.086	
Peak 2	13.6150983	79.0743561	60.899	
Peak 3	13.6150983	103.110565	60.9658	
Peak 4	2.26449667	104.113853	60.0266	
Peak 5	1.95884833	118.084915	100.0772	82.0911
Peak 6	13.4163567	135.09996	88.8777	68.9882
Peak 7	1.92302667	138.053299	138.0015	
Peak 8	13.4553233	199.167007	111.1103	68.8976
Peak 9	2.24256167	207.204376	103.9745	59.9237
Peak 10	10.9135883	282.276062	265.2249	247.1913
Peak 11	13.4163567	287.218414	199.0778	111.0254
Peak 12	1.86624	325.109375	288.8931	126.9222
Peak 13	10.42607	330.332947	312.3414	101.9587
Peak 14	10.84576	356.348328	338.3103	101.9976
Peak 15	1.88771167	360.146133	324.9626	288.9922
Peak 16	12.4952333	391.279999	148.9516	121.03

	BE4 (Sample 4)			
	RT	MS1(m/z)	MS2(m/z)	MS3(m/z)
Peak 1	13.9882783	79.0743332	60.9917	
Peak 2	13.9882783	103.110535	60.9893	
Peak 3	2.225215	104.113823	104.0125	
Peak 4	1.973025	130.084671	83.9342	
Peak 5	12.5871583	135.099915	88.9249	59.0415
Peak 6	1.93799333	138.053238	137.9303	
Peak 7	12.5871583	199.166946	111.1525	68.9211
Peak 8	1.78979667	265.082336	135.9724	118.9279
Peak 9	10.9211317	282.276001	265.21	247.3089
Peak 10	12.5871583	287.218353	199.0962	111.0785
Peak 11	10.35178	330.332886	312.3331	102.0276
Peak 12	12.893235	338.337769	321.2432	303.2547
Peak 13	10.79307	356.348236	338.3278	102.0184
Peak 14	12.5029767	391.279877	148.9346	120.9989

	BE5 (Sample 5)			
	RT	MS1(m/z)	MS2(m/z)	MS3(m/z)
Peak 1	12.9643917	61.0639496	61.0828	
Peak 2	12.9643917	79.0743408	61.0057	
Peak 3	13.736335	103.11055	60.9534	
Peak 4	2.25353833	104.113838	103.9469	59.8176
Peak 5	1.91789167	130.084686	83.9571	55.7687
Peak 6	11.8570167	135.09993	88.9895	
Peak 7	1.91789167	138.053284	137.9933	94.2974
Peak 8	5.54421833	144.0821	97.9591	70.0993
Peak 9	1.91789167	144.100174	97.9591	70.0993
Peak 10	1.98940333	144.100204	97.9591	70.0993
Peak 11	13.9460417	199.166962	111.209	68.9239
Peak 12	1.80480333	265.082397	118.8564	72.869
Peak 13	10.9146033	282.276031	265.1783	247.2656
Peak 14	11.8570167	287.218384	191.0818	110.9977
Peak 15	10.392585	330.332916	312.2337	102.0625
Peak 16	10.844945	356.348297	338.3009	102.0603
Peak 17	1.86457	360.146118	324.9405	144.9609
Peak 18	12.5077717	391.279907	148.9234	120.8753

	BE8 (Sample 8)			
	RT	MS1(m/z)	MS2(m/z)	MS3(m/z)
Peak 1	12.4732167	61.0639458	61.2996	
Peak 2	12.4732167	79.0743408	60.8827	
Peak 3	13.3664667	103.110542	60.8843	
Peak 4	13.4815067	135.09993	88.9332	69.026
Peak 5	13.5380467	136.987152	108.8667	80.9309
Peak 6	13.6813517	136.99585	108.8667	80.9309
Peak 7	1.26978833	137.068016	108.8667	80.9309
Peak 8	2.50825333	137.094315	108.8667	80.9309
Peak 9	12.1777017	137.094315	108.8667	80.9309
Peak 10	3.84857667	137.09436	108.8667	80.9309
Peak 11	2.47193333	152.045029	123.9966	94.9504
Peak 12	2.490035	153.05278	124.9418	92.8934
Peak 13	7.695655	153.089088	124.9418	92.8934
Peak 14	5.49266333	153.089462	124.9418	92.8934
Peak 15	8.411095	153.125488	124.9418	92.8934
Peak 16	2.490035	167.032074	151.975	124.0598
Peak 17	12.9475283	199.166962	111.1023	68.953
Peak 18	10.9061183	282.276001	265.1479	247.2551
Peak 19	12.0883467	287.218384	199.0222	111.1176
Peak 20	11.7407283	323.254486	247.1617	121.0678
Peak 21	12.4732167	323.254486	247.1617	121.0678
Peak 22	10.3812133	330.332886	312.3016	102.0582
Peak 23	13.9496883	351.325363	275.2528	121.0859
Peak 24	10.9061183	356.348236	338.3432	101.9526
Peak 25	13.4815067	375.321442	357.3036	339.3403
Peak 26	14.000285	377.337036	319.3293	235.2205
Peak 27	11.661945	403.352509	385.2427	367.3871
Peak 28	12.964395	405.368073	387.3098	369.3398
Peak 29	12.4732167	431.383453	413.4841	395.2727
Peak 30	12.8286367	447.378082	389.322	371.2492

Table S1. LC-MS profiling using MZmine of bioactive bean extracts BE2, BE4, BE5, and BE8: Retention time (RT, min), MS^1, MS^2 and MS^3 (*m/z*).

A

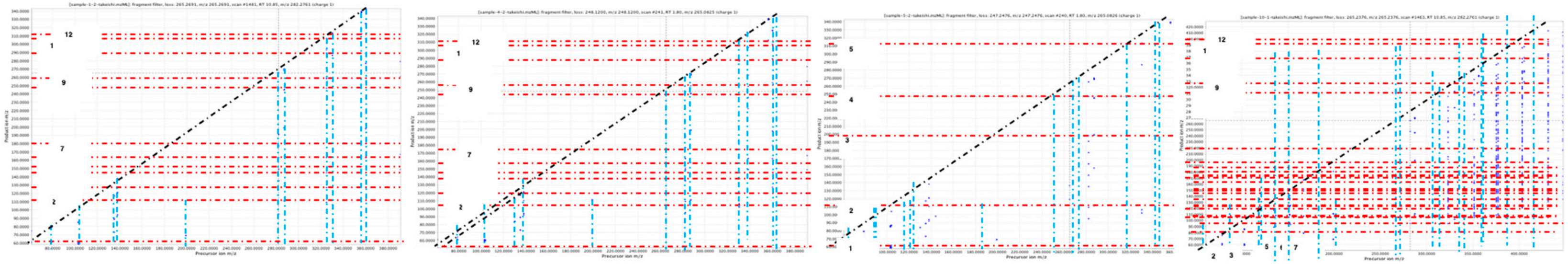

Represent the diagnostic product ions that are required to be considered a putative metabolites x

Represent the [M + H]+ precursor ions.

B

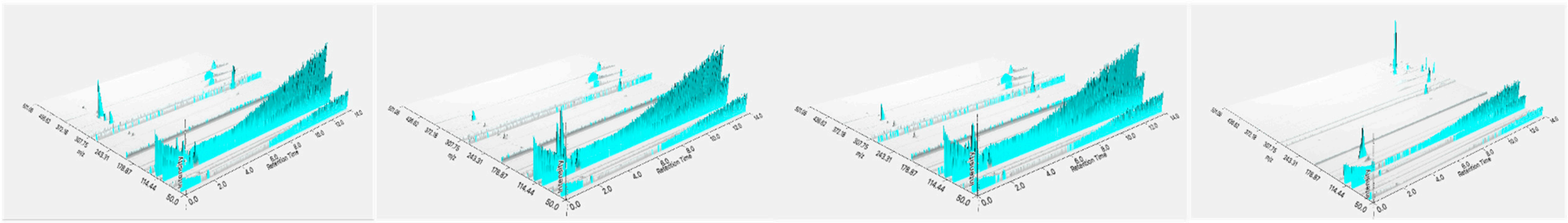

BE2 BE4 BE5 BE8

Figure S4. LC-MS profiling of bioactive bean extracts. (A) Diagnostic Fragmentation Filtering (DFF) plot for metabolites analysis. (B) 3D visualization of MS data.

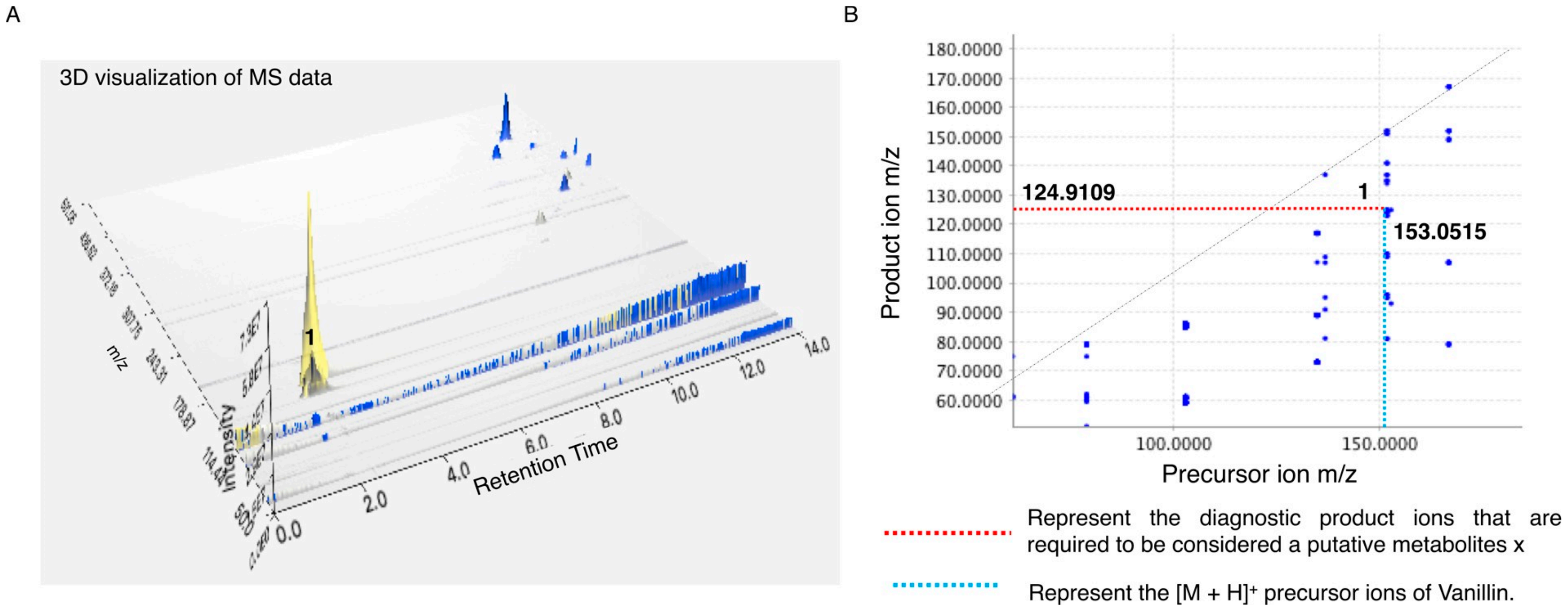

Figure S5. (A) Three-dimensional (3D) liquid chromatography/mass spectrometry (LC-MS) comparison of bean extract 8 (BE8) and vanillin. (B) Diagnostic fragmentation filtering (DFF) plot of the vanillin in BE8.

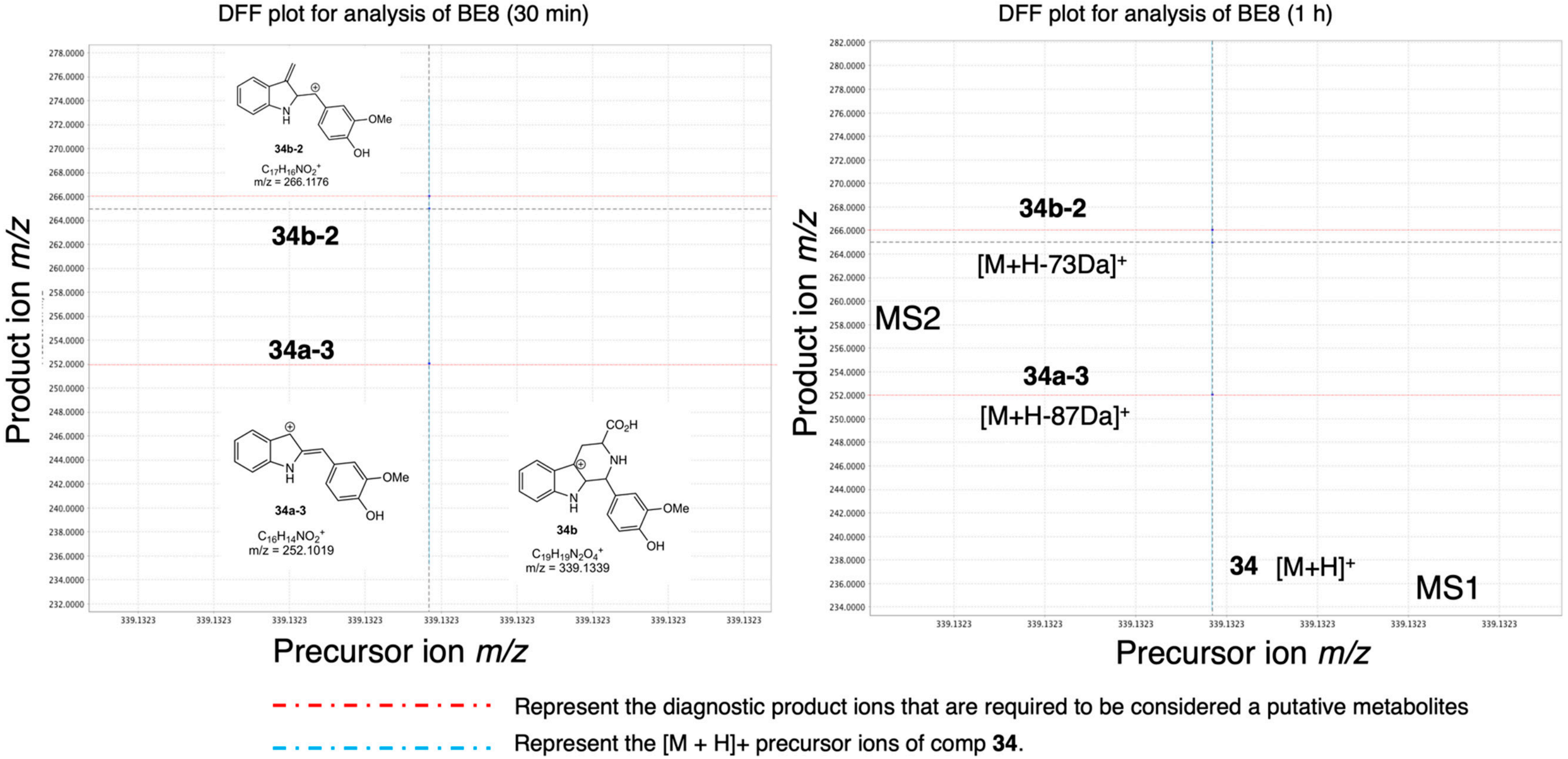

Figure S6. Diagnostic fragmentation filtering (DFF) identification of β-carboline alkaloid **34**: DFF analysis of **34** in modified vanilla BE -8 mixture after 30 min and 1 h of treatment.

Table S2. List of primers.

Gene	Primer sequences (5’ → 3’)			references:
ATGL	F: ACCAGCATCCAGTTCAACCT	R: ATCCCTGCTTGCACATCTCT	-->	Adipose triglyceride lipase expression in human adipose tissue and muscle. Role in insulin resistance and response to training and pioglitazone
DGAT1	F: TATTGCGGCCAATGTCTTTGC	R: CACTGGAGTGATAGACTCAACCA	-->	https://www.hindawi.com/journals/omcl/2018/8515343/tab1/
SREBP1	F: CAGCCCACTTCATCAAGG	R: ACTGTTGCCAAGATGGTTCCG	-->	https://www.hindawi.com/journals/omcl/2018/8515343/tab1/
GAPDH	F: GAAGGTGAAGGTCGGAGTC	R: GAAGATGGTGATGGGATTTC	-->	Bhullar, K.S., Shang, N., Kerek, E. et al. Mitofusion is required for MOTS-c induced GLUT4 translocation. Sci Rep 11, 14291 (2021). https://www.nature.com/articles/s41598-021-93735-2
